# Late-stage pregnancy toxemia in does: biochemical, hormonal, and histopathological changes

**DOI:** 10.1007/s11250-026-04952-8

**Published:** 2026-03-24

**Authors:** Mahmoud H. Emam, Magdy M. Elgioushy, Maha M. Elgebaly, Amal M. Aboelmatty, Heba Gouda

**Affiliations:** 1 Department of Animal Medicine, Division of Internal Medicine, Faculty of Veterinary Medicine, Zagazig University, Zagazig, 44511 Egypt; 2 Department of Animal Medicine, Division of Internal Medicine, Faculty of Veterinary Medicine, Aswan University, Aswan, 37916 Egypt; 3 Department of Animal Reproduction and AI, Veterinary Research Institute, National Research Centre, Giza, 12622 Egypt; 4 Department of Theriogenology of Faculty of Veterinary Medicine, Zagazig University, Zagazig, 44511 Egypt

**Keywords:** Pregnancy toxemia, Does, Liver, Histopathology, BHBA

## Abstract

Pregnancy toxemia (PT) in does represents a major economic concern, leading to significant financial losses due to increased mortality, decreased reproductive performance, and the high costs of treatment and management. Our study aimed to investigate the biochemical, hormonal, and histopathological changes during the late stage of PT in pregnant does. Seventeen (17) does diagnosed with PT were clinically, biochemically evaluated and compared to a control group of seven healthy pregnant does (C1). Additionally, four liver samples from healthy non-pregnant goat (C2) were used as histopathological control. Clinical, biochemical, and hormonal parameters were assessed, and statistical analysis was conducted using the t-test to evaluate differences between the groups. The results revealed significant alterations in progesterone and estrogen levels, beta hydroxy butyric acid (BHBA), non-esterified fatty acids (NEFA), liver enzyme activity, cholesterol, glucose, creatinine concentrations, and acute phase proteins. Histopathological examination of liver tissues also showed marked differences between the PT and healthy non- pregnant does (C2). However, oxidative stress biomarkers did not demonstrate significant discriminatory power between healthy and PT-affected does. These findings suggest that biochemical indicators, and histopathological liver changes are reliable markers for diagnosing late-stage pregnancy toxemia in does.

## Introduction

Pregnancy toxemia (PT) is a life-threatening metabolic disorder occurring in small ruminants during the late stage of gestation (Ji et al. 2023). This disorder develops mainly due to a negative energy balance, where the dam fails to satisfy the heightened energy requirements of late pregnancy. Fetal development leads to a reduction in rumen capacity, which may result in inadequate feed intake (Uztimur and Ünal 2024). The high nutritional demands during late gestation are due to nearly 80% of fetal growth occurs in the last six weeks of pregnancy (Rook 2000). During this critical period, the fetoplacental unit depends on glucose and lactate as energy sources, utilizing approximately 30–40% of the maternal glucose production (Rook 2000). Pregnant sheep or goats carrying more than one fetus are vulnerable to this disorder because their ability to ingest adequate feed is markedly reduced (Ermilio and Smith 2011). Additionally, weak multiparous ewes in their final trimester are also at increased risk (Simões and Margatho 2024). Decreased dietary energy level is also a predisposing factor (Edmondson and Pugh 2009), as well as a genetic propensity that is more prevalent in sheep than goats (Moallem et al. 2012). Other risk factors are associated with the development of ovine pregnancy toxemia including thin and over-conditioned ewes (Crilly et al. 2021). Also, concomitant diseases such as Johne’s disease, GIT nematodes, and liver flukes increase the risk of the development of ovine pregnancy toxemia (Papadopoulos et al. 2013; Barbagianni et al. 2015).

Adipose tissue is mobilized to provide energy for fetal growth, generating ketone bodies such as acetoacetate, acetone, and β-hydroxybutyric acid (BHBA) (Moghaddam and Hassanpour 2008; Singh et al. 2022). By inhibiting hepatic gluconeogenesis, both excessive lipolysis (Guo et al. 2020) and high BHBA levels may exacerbate maternal hypoglycemia (Schlumbohm and Harmeyer 2008). Although, the disease has a very low morbidity rate, if therapy is delayed, the fatality rate can be quite high (Simpson et al. 2019), resulting in significant economic impacts due to therapeutic costs and the loss of dams and fetuses (Rook 2000). Previous studies (Rook 2000; Lima et al. 2016) have shown high case fatality rates in goats affected by PT exceeding 80% in untreated animals. The course of untreated PT has been reported to range from 12 h to one week, typically between three and four days (Smith and Sherman 2009; Lima et al. 2012).

Pregnancy toxemia can present in either clinical or subclinical forms according to presence or absence of clinical signs, not the level of BHB. The subclinical form is characterized by hyperketonemia with serum BHBA ranging from 0.8 to1.6 mmol/L without clinical signs. In contrast, the clinical form is indicated by elevated levels of blood ketone bodies (serum > 1.6 mmol/L) accompanied by clinical signs such as decreased appetite, nervous manifestations and in severe cases, coma, and death (Vasava et al. 2016; Xue et al. 2019). Other previous studies reported that the BHB cut-off for mild or moderate hyperketonemia (subclinical PT) is above 0.86 mmol/L, whereas BHB concentration above 3.0 mmol/L is indicative of severe and clinical hyperketonemia and clinical PT (Fiore et al. 2021; Balikci et al. 2009). Biochemical markers are valuable tools for diagnosing PT; elevated BHBA levels and hypoglycemia are reliable indicators of the condition in ewes and does (Fthenakis et al. 2012; Cal-Pereyra et al. 2015; Iqbal et al. 2022). It is widely accepted that ketone bodies can alter oxidative stress markers due to their generation of harmful free radicals (e.g., superoxide, hydroxyl). These radicals can cause lipid peroxidation, damaging cell membranes and compromising their function (Al-Qudah 2011).

Prompt identification of PT in high-risk animals is crucial to ensure timely therapeutic and preventive intervention, as the mortality rate among affected animals is high (Andrews 1997). Pregnancy toxemia can be reliably diagnosed based on the history, clinical signs, hematological indicators, and serum biochemical analyses (Lima et al. 2016). However, pregnancy toxemia in does is a complex metabolic disorder with a poor prognosis, particularly in the late stage of the disease. Therefore, a better understanding of the pathophysiology of pregnancy toxemia in does is critical to reduce its negative effects. Accordingly, the main objective of this study was to investigate the biochemical, hormonal, and histopathological changes associated with this disorder.

## Materials and methods

### Animals and history

A total of seventeen (17) pregnant Zaraibi does weighing 20–30 kg were admitted and examined at Obstetrics and Gynecology Department, Faculty of Veterinary Medicine Zagazig University between November 2024, and March 2025. These female goats were brought in with the main complaints of weakness, inability to stand, anorexia, and sternal recumbency. Also, a history of imminent or overdue parturition was reported. All cases were carrying more than one fetus, as confirmed by ultrasonography. The owners of the affected cases lacked sufficient records of deworming and vaccination protocols. Urine samples were checked with urine strips to confirm the hypoglycemia and ketosis status. A total of 7 apparently healthy pregnant goats, belonging to the Faculty of Veterinary Medicine Farms, were used as the control group. All goats in the control group were in late gestation (imminent parturition) and were carrying multiple fetuses.

## Clinical examination

**Fig. 1:**
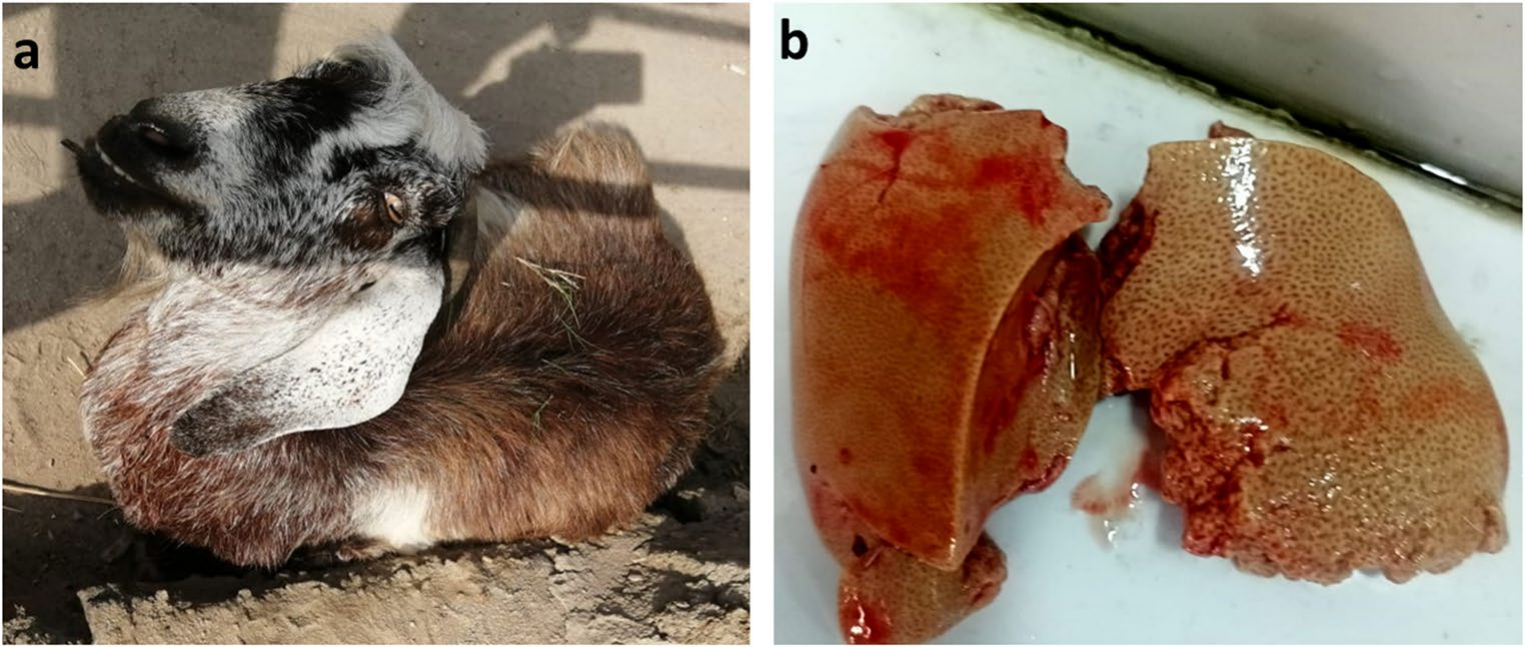
Recumbent pregnant doe showing nervous signs including blindness, incoordination, ataxia, and muscle tremors (**a**). Postpartum liver part from PT doe enlarged with fatty liver appearance (**b**)

Pregnancy toxemic and control does in this study were subjected to thorough clinical examinations following the recommendations of Jackson et al. (2002). The clinical examination included measuring rectal temperature, assessing respiratory rate, inspecting the mucous membranes, auscultating the heart rate, and evaluating ruminal contractions. The affected does were thoroughly examined for nervous signs including blindness, incoordination, ataxia, and muscle tremors. The body condition score (BCS) of all cases was assessed using a scale ranging from 1 (very lean) to 5 (very obese), as described by Ghosh et al. (2019). Assessment was based on both visual inspection and palpation to evaluate the amount of muscle, fat, and connective tissue. Considering the clinical history, physical examination, and urine strip test results, the affected cases were diagnosed as pregnancy toxemia. **(**Fig. 1**).**

## Serum collection and biochemical analysis

Five milliliters of blood (5 ml) were collected into plain tubes (without anticoagulant) for serum preparation. Samples were centrifuged at 3000 rpm for 20 min within two hours of collection to obtain serum. The clear supernatant was carefully aspirated using sterile disposable Pasteur pipettes and transferred into sterile 1.5 ml Eppendorf tubes. Serum aliquots were stored at − 20 °C until biochemical analyses were conducted. Non-esterified fatty acid (NEFA; mmol/L) concentrations were determined using photometric kits (DIA Lab, Austria), while β-hydroxybutyrate (BHBA) levels were assessed with commercial kits from POINTE Scientific Inc. (USA). Serum levels of estrogen (pg/mL), progesterone (ng/mL), catalase (CAT; U/L), glutathione peroxidase (GPX; mU/mL), superoxide dismutase (SOD; U/mL), nitric oxide (NO; µmol/L), total antioxidant capacity (TAC; mM/L), total protein (g/dL), albumin (g/dL), globulin (g/dL), alanine aminotransferase (ALT; U/L), aspartate aminotransferase (AST; U/L), creatinine (mg/dL), glucose (mg/dL), total cholesterol (mg/dL), and zinc (µg/dL) were quantified using a Beckman AU5800 analyzer (Beckman Coulter, California, USA). The analyzer was calibrated before each run using standard assay kits. All analysis followed the manufacturer’s protocols.

## Histopathological examination

Four liver tissue samples were obtained from governmental abattoir from healthy non pregnant goats (serving as control). Although all PT cases resulted in death, liver samples were obtained and analyzed from only seven cases, as permission was granted by their owners. The specimens were fixed in 10% neutral buffered formalin for 72 h, then rinsed under running tap water. They were subsequently dehydrated through graded concentrations of ethanol, cleared using Histo-Choice^®^ (Sigma-Aldrich, St. Louis, USA), infiltrated, and embedded in paraffin wax. Sections of 5 μm thickness were cut, stained with hematoxylin and eosin, and examined under a light microscope. Images were taken by a Leica microscope provided with a camera (Leica Microsystems Inc., Buffalo Grove, IL). All histopathological techniques followed the instructions of Suvarna et al. (2019). The degree of hepatic steatosis was evaluated according to Table 1.

**Table 1:** Hepatic steatosis scores and severity based on the percentage of hepatocytes that contained lipid vacuoles “fat cells”

Legend	Severity	Description	Score
A	Absent	< 5%	0
B	Mild	5–12%	1
C	Marked	12–33%	2
D	Severe	> 60%	3

### Statistical analysis

All statistical analyses were conducted using SPSS software (version 20.0, SPSS Inc., USA). Data normality was evaluated using the Shapiro–Wilk W test, confirming a normal distribution. Comparisons of biochemical and hormonal variables were performed using Welch, Brown-Forsythe, and independent samples t-test, and results are presented as mean ± SE. Statistical significance was determined at probability thresholds of *P* < 0.05 and *P* < 0.01.

## Results

### Descriptive statistics of clinical parameters

Mean ± standard deviation (SD) of rectal body temperature, respiratory rate, heart rate, body condition score (BCS), gestation length, and number of fetus in control and PT does were documented in Table 2. Also, clinical parameters in control and pregnancy toxemic (PT) does including appetite status, defecation, systemic disturbance, pain reaction, ruminal contraction, mucous membrane, nervous signs as well as recumbency status and the percentage of the dead cases are documented in Table 3.

**Table 2:** Mean ± standard deviation (SD) of rectal body temperature, respiratory rate, heart rate, body condition score (BCS), gestation length, and number of fetus in control and PT does

Variables	Control	PT	*P* value
Body temperature (°C)	39.4 ± 0.3	38.1 ± 0.8	**< 0.001**
Respiratory rate (cycle/ min)	24.4 ± 3.4	32.8 ± 5.1	**< 0.001**
Heart rate (beats/ min)	82.6 ± 4.6	94.0 ± 8.1	**< 0.001**
BCS	3.3 ± 0.2	2.4 ± 0.3	**< 0.001**
Gestation length (days)	124 ± 6.7	130 ± 12.9	0.2
Number of fetus	2.2 ± 0.4	2.6 ± 0.5	0.06

**Table 3:** Clinical parameters in control and pregnancy toxemic (PT) does

Items	Control (*n* = 7)		PT (*n* = 17)	
	*N*	%	*N*	%
Appetite Inappetence Anorexia	2 0	28.6 0	7 10	41.2 58.8
**Defecation status** Normal Scanty Diarrhea	7 0 0	100 0 0	7 8 2	41.2 47.1 11.7
Systemic disturbance*	0	0	17	100
Pain reaction**	0	0	11	64.7
**Nervous signs**	0	0	9	52.9
**Recumbency**	0	0	10	58.8
**Mucous membrane** Normal Pale	7 0	100 0	5 12	29.4 70.6
Rumen contraction*** Normal Hypomotility	5 2	71.4 28.6	0 17	0 100
**Death**	0	0	17	100

Systemic disturbance*: mean elevation of body TH, respiratory rate, heart rate above the control level; Pain reaction**: include expiratory grunting, stiffness in gait and arched back; Rumen contraction***: the ruminal contraction (2–5 every 2 min were considered normal otherwise it was considered hypomotility

## Hormonal and oxidative stress analysis

A significant increase in the level of estrogen hormone was reported in PT cases compared to the control (*P* = 0.004). Conversely, a significant reduction in the concentration of progesterone hormone in PT does compared to the healthy ones (*P* = 0.006). The mean values of estrogen (pg/ml) and progesterone (ng/ml) were (92.44 ± 33.49, 124.27 ± 17.08) and (8.63 ± 1.86, 6.17 ± 2.31) for control and PT cases, respectively. We did not detect any significant differences at the level of Catalase (CAT) enzyme (*P* = 0.7), Glutathione peroxidase (Gpx) (*P* = 0.21), Total antioxidant capacity (TAC) (*P* = 0.48), Superoxidase enzyme (SOD) (*P* = 0.26), and Nitric oxide (NO) (*P* = 0.62) between control and PT cases **(**Table 4**).**

**Table 4:** Mean values ± SD of hormonal and oxidative stress biomarkers in control and pregnancy toxemia (PT) does

Items	Control (*n* = 7)	PT (*n* = 17)	*P* value
Estrogen (pg/ml)	92.44 ± 33.49	124.27 ± 17.08	**0.004**
Progesterone (ng/ml)	8.63 ± 1.86	6.17 ± 2.31	**0.006**
CAT^1^ (U/L)	139.09 ± 61.21	212.03 ± 123.98	0.7
GPX^2^ (mU/mL)	894.73 ± 154.83	808.64 ± 188.95	0.21
TAC^3^ (mM/L)	0.32 ± 0.03	0.31 ± 0.05	0.48
SOD^4^ (U/ml)	337.50 ± 39.17	360 ± 58.09	0.26
NO^5^ (µmol/L)	39.04 ± 7.32	40.35 ± 6.43	0.62

^1^CAT: Catalase enzyme; ^2^GPX: Glutathione peroxidase; ^3^TAC: total oxidant capacity; ^4^SOD: superoxidase; ^5^NO: Nitic oxide

### Biochemical analysis

Serum concentrations of NEFA and BHBA were markedly elevated in does with pregnancy toxemia compared to healthy controls, with both parameters showing highly significant differences (*p* < 0.001). The mean ± SD values of NEFA (mmol/L) were 0.9 ± 0.2 in PT cases and 0.3 ± 0.06 in controls, while BHBA (mmol/L) averaged 1.7 ± 0.2 in PT does versus 0.5 ± 0.2 in controls. Serum total protein (TP) and globulin concentrations were also significantly higher in PT does (*p* < 0.001 for both), with TP values of 3.2 ± 0.20 g/dL versus 2.7 ± 0.20 g/dL, and globulin levels of 1.8 ± 0.28 g/dL versus 1.3 ± 0.37 g/dL for PT and control animals, respectively. Conversely, the albumin-to-globulin (A/G) ratio was significantly reduced in PT cases (*p* = 0.01), averaging 0.80 ± 0.28 compared to 1.3 ± 0.72 in controls, whereas serum albumin alone showed no significant difference (*p* = 0.64). Liver enzyme activities were notably increased in affected does, with ALT (U/L) and AST (U/L) levels significantly higher than controls (*p* < 0.001 and *p* = 0.03, respectively), recording mean ± SD values of 4.5 ± 2.5 and 18.21 ± 7.5 for PT cases versus 0.92 ± 0.67 and 12.97 ± 1.45 for controls. All results are documented in Table 5.

**Table 5:** Mean values ± SD of serum biochemical parameters in control and pregnancy toxemia (PT) does

Items	Control (*n* = 7)	PT (*n* = 17)	*P* value
NEFA^1^ (mmol/L)	0.3 ± 0.06	0.9 ± 0.2	**< 0.001**
BHBA^2^ (mmol/L)	0.5 ± 0.2	1.7 ± 0.2	**< 0.001**
Total proteins (g/dl)	2.7 ± 0.20	3.2 ± 0.20	**< 0.001**
Globulin (g/dl)	1.3 ± 0.37	1.8 ± 0.28	**< 0.001**
Albumin (g/dl)	1.44 ± 0.42	1.37 ± 0.31	0.64
A/G ratio^3^ (%)	1.3 ± 0.72	0.80 ± 0.28	**0.01**
ALT^4^ (U/L)	0.92 ± 0.67	4.5 ± 2.5	**< 0.001**
AST^5^ (U/L)	12.97 ± 1.45	18.21 ± 7.5	**0.03**
Creatinine (mg/dl)	1.6 ± 0.12	1.8 ± 0.24	**0.04**
Glucose (mg/dl)	52.5 ± 6.3	78.1 ± 4.8	**< 0.001**
Total cholesterol (mg/dl)	50.34 ± 5.86	41.74 ± 1.85	**< 0.001**
Zinc (µmol/L)	22.73 ± 3.9	25.34 ± 6.4	0.23

^1^NEFA: Non-esterified fatty acid; ^2^BHBA: Beta hydroxy butyric acid; ^3^A/G ratio: Albumin/ globulin ratio; ^4^ALT: Alanine transaminase; ^5^AST: Aspartate transaminase

Serum creatinine and glucose levels were significantly elevated in does affected by pregnancy toxemia compared to healthy controls, with p-values of 0.04 and < 0.001, respectively. The mean ± SD values of creatinine (mg/dL) were 1.8 ± 0.24 for PT cases versus 1.6 ± 0.12 for controls, while glucose (mg/dL) averaged 78.1 ± 4.8 in PT does compared to 52.5 ± 6.3 in controls. In contrast, total cholesterol concentrations were markedly reduced in PT animals (*p* < 0.001), with mean ± SD values of 41.74 ± 1.85 mg/dL versus 50.34 ± 5.86 mg/dL in controls. Serum zinc (µmol/L), however, showed no significant variation between PT does and healthy counterparts (*p* = 0.23). All results are documented in Table 5.

### Histopathological changes

**Fig. 2:**
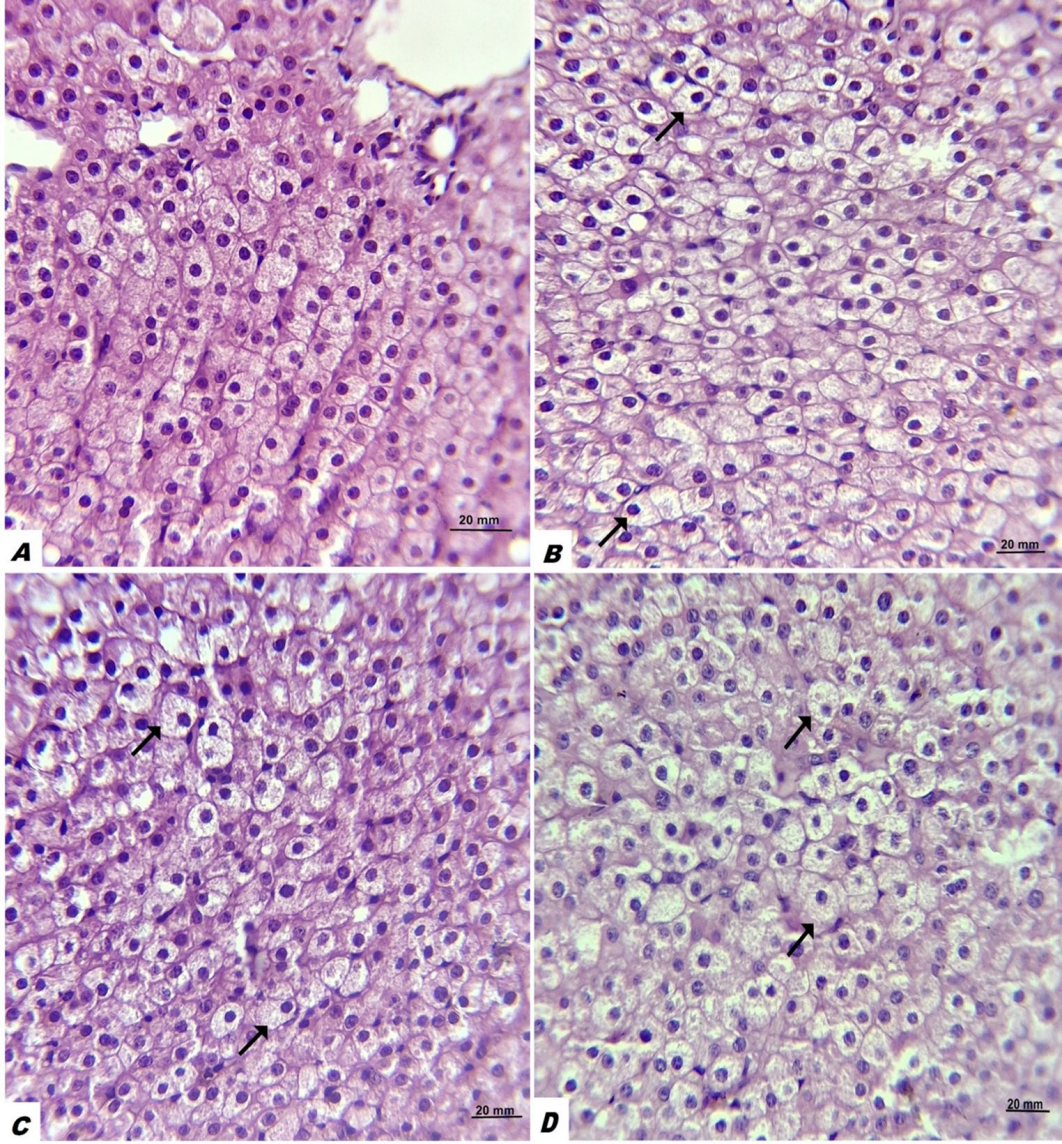
Liver with varying degrees of hepatic steatosis with presence of lipid vacuoles inside hepatocytes pushing the nucleus of hepatocytes to the periphery giving signet ring appearance. **A**: no hepatic steatosis; **B**: mild hepatic steatosis; **C**: moderate hepatic steatosis; **D**: severe hepatic steatosis

Regarding the histopathological changes, the liver samples from PT cases that died revealed that excessive accumulation of fat cells (lipid vacuoles) were detected inside hepatocytes. Varying degrees of hepatic steatosis with presence of lipid vacuoles inside hepatocytes pushing the nucleus of hepatocytes to the periphery gives the classical signet ring appearance (Fig. 2). Out of 7 liver samples examined for PT does, 71.4% (5/7) exhibited severe hepatic steatosis and 28.6% (2/7) exhibited marked hepatic steatosis. Among the 4 control healthy samples, 75% (3/4) revealed absent hepatic steatosis (Score 0), and 25% (1/4) showed moderate hepatic steatosis **(**Fig. 2**).**

## Discussion

Despite being extensively studied, data on the clinical, biochemical, and histopathological changes associated with late-stage pregnancy toxemia (PT) in does remain limited. Therefore, our work aimed to monitor these changes. In affected does, PT manifested with a variety of clinical signs; key findings included anorexia, scant feces, and pain reactions characterized by expiratory grunting, stiffness in gait, and an arched back. These findings align with those reported by Gaadee and Gehan in ewes with PT (Gaadee and Gehan 2021). The ruminal hypomotility seen in all does with PT in this study may be attributed to malnourishment in pregnant dams that might result in the formation of ketone bodies, impacting feed intake, ruminal contractions, and body condition (Andrade et al. 2019).

Interestingly, in this study, estrogen concentrations were higher than normal, whereas progesterone levels were lower than normal. These findings differ from previous reports, which indicated that progesterone levels tend to be elevated and that estrogen concentrations increase primarily prior to parturition, particularly in cases of multiple fetuses (Rawlings and Ward 1977). Progesterone is crucial for maintaining pregnancy as it promotes uterine quiescence and prepares the mammary glands for milk production (Amin and Ibrahim 2010). Additionally, the rise in estrogen levels during late pregnancy, particularly before parturition, influences the development of the cervix and other physiological changes associated with preparing for delivery (Khanum et al. 2008). Other studies concluded that, in late pregnancy, a high progesterone-to-estrogen ratio is associated with a higher risk of toxemia (Yahi et al. 2017). In our study, disruption of this hormonal equilibrium could exacerbate metabolic problems and increase the risk for PT. Also, we propose that elevated estrogen levels may contribute to pregnancy toxemia by reducing maternal blood glucose, particularly during late gestation. Although there is limited data about the association between estrogen and blood glucose in small ruminants, several studies in human medicine have reported that elevated estrogen levels suppress hepatic glucose production and have an anti-diabetic effect (Yan et al. 2019). However, our findings related to alterations in reproductive hormone balance should be interpreted with caution, and further studies are required to understand the underlying mechanisms.

Significant differences were detected in the concentrations of BHBA and NEFA between control and PT goats. These findings were expected, as previous studies have reported that goats with PT experience excessive fat mobilization, resulting in elevated ketone body concentrations to meet the energy demands of fetal growth (Moghaddam and Hassanpour 2008; Guo et al. 2020; Singh et al. 2022). Interestingly, despite the affected cases being in a late stage of the disease and exhibiting nervous signs, the mean BHBA concentration in the PT group was only 1.7 mmol/L. This BHBA cutoff value contradicts previous reports indicating that clinical pregnancy toxemia is typically associated with BHBA levels > 3 mmol/L, thereby highlighting the importance of clinical signs, in addition to biochemical parameters, for the accurate diagnosis and prognosis of pregnancy toxemia. The increased activities of AST and ALT noted in the does with PT of our investigation are align with Vasava et al. (2016), who reported a similar increase of these enzymes in pregnancy toxemic goats. Also, Iqbal et al. (2022) attributed this increase to hepatic damage or hepatic lipidosis, resulting from fat mobilization and severe fatty accumulation. Serum creatinine was significantly higher in PT does compared to controls, and this may be considered an indicator of the involvement of the kidneys due to increased catabolism and severe kidneys dysfunction (Souza et al. 2020). On the other hand, our findings contradicted those of Tharwat and Al-Sobayil (2014), who found no association between renal functions and pregnancy toxicity.

Hypoglycemia is widely recognized as one of the most common biochemical indicators of this disease (Kelay and Assefa 2018). A higher energy requirement in late pregnancy, particularly with twins or triplets, along with a lack of sufficient energy, leads to this hypoglycemia (Vasava et al. 2016). However, the significant increase in serum glucose levels observed in does with PT in this study contradicts the findings of several previous studies on both ewes (Gaadee and Gehan 2021) and does (Vasava et al. 2016), which reported hypoglycemia in affected PT animals. However, Iqbal, Souto, and their colleagues (Iqbal et al. 2022; Souto et al. 2013) observed hyperglycemia in severe cases of ovine PT with poor prognosis, which aligns with our findings. According to Marteniuk and Herdt (1988), blood glucose levels change as PT progresses, beginning lower in the early stages and increasing to hyperglycemia during coma, which frequently occurs after fetal death. This hyperglycemia may be attributed to stress conditions that elevate cortisol levels. Cortisol is known for its gluconeogenic properties and its inhibitory action on insulin, which disrupts peripheral receptors, impairs insulin utilization, and interferes with the glycolytic process (Schlumbohm and Harmeyer 2008). Additionally, Moallem et al. (2012) concluded that the decreased plasma insulin concentrations during late pregnancy, occurring with an increased number of fetuses, are contributing factors to hyperglycemia in PT does.

The significant reduction in total cholesterol in does affected by pregnancy toxemia compared to controls is consistent with the observations of Gaadee and Gehan (2021), who reported a similar decline in cholesterol levels in ewes with this condition. This reduction may result from impaired hepatic function, limiting the liver’s capacity to export fat as VLDL and promoting hepatic lipid accumulation (Grummer 1993). Furthermore, hyperketonemia associated with elevated energy demands may enhance fat mobilization, producing notable alterations in the lipid profile (Rook 2000; Bani Ismail et al. 2008). This reduction in cholesterol levels can be ascribed to diminished feed intake, liver dysfunction, and physiological changes in the endocrine system (Waziri et al. 2010). In contrast, a significant increase in total cholesterol levels was reported of ewes with experimental PT (Xue et al. 2019), and a Rembi ewe with PT (Aiche et al. 2023). The increased cholesterol level was linked to diminished peripheral tissue responsiveness to insulin, resulting in increased lipolysis and subsequently elevated blood cholesterol levels (de Souza et al. 2019).

Although significant differences were observed in the mean values of total proteins and non- significant differences in albumin levels between healthy and PT does, the average concentrations of both parameters in both groups were lower than the commonly accepted clinical reference ranges for healthy goats. These findings suggest that total protein and albumin may be affected by pregnancy status itself, regardless the presence of PT. Similar to our findings, previous studies have reported no significant changes in plasma total protein and albumin levels in does with pregnancy toxemia (PT) (Uzti̇mür and Ünal 2024). However, the significant hyperglobulinemia observed in does with PT is in accordance with the findings of Tharwat and his colleagues (Tharwat et al. 2025) in ewes and does with PT. This notable increase in globulin in ewes with PT was attributed to a strong inflammatory response triggered by pregnancy toxemia (Darwish and El-Ebissy 2019).

Oxidative stress is not a classical disease and therefore lacks a distinct clinical presentation (Fayed et al. 2018). Previous reports indicate that ruminants may undergo oxidative stress during the transition period, which can predispose them to periparturient disorders and metabolic diseases (Rook 2000). In the present study, no significant differences were observed in the activities of catalase (CAT), glutathione peroxidase (GPx), total antioxidant capacity (TAC), superoxide dismutase (SOD), or nitric oxide (NO) levels between healthy and pregnancy toxemic does. These findings suggest that oxidative stress is a common physiological challenge during late gestation in goats, regardless pregnancy toxemia status. Conversely, previous study revealed a significant increase in malondialdehyde (MDA) levels, accompanied by significant reductions in glutathione (GSH), glutathione peroxidase (GPx), catalase (CAT), and superoxide dismutase (SOD) in pregnancy toxemia (PT) does compared with healthy controls. Further studies with a larger number of pregnant does are required to validate our observation.

Pregnancy toxemia in does is typically confirmed through necropsy, as early diagnosis remains difficult. Livers of affected animals are characteristically enlarged, yellow, and fragile, likely due to progressive triglyceride accumulation (Xue et al. 2019). Histopathological examination of liver samples from PT cases that died demonstrated marked intracellular fat deposition within hepatocytes. These findings are consistent with Ji et al. (2023), who reported increased nucleolar prominence, proliferation of cytoplasmic glycogen granules, and extensive lipid replacement of hepatocytes accompanied by severe vacuolization. Our hepatic histopathological findings should be interpreted with caution because the control liver samples were obtained from non- pregnant goats at the abattoir.

## Conclusion

The current study concluded that alterations in biochemical parameters, including non-esterified fatty acids (NEFA), β-hydroxybutyrate (BHB), liver enzymes, glucose, creatinine, and total cholesterol, serve as reliable indicators of the advanced stages of pregnancy toxemia in does. However, the observed alterations in reproductive hormone levels require further investigation to clarify the mechanisms regulating these hormonal changes. Although histopathological changes were observed in the liver samples of PT cases compared to controls, these findings should be interpreted with caution, as the control liver samples were obtained from non-pregnant goats. Improving the detection and prediction efficacy of metabolic disorders such as PT can facilitate improved management practices, targeted therapeutic interventions, and a deeper understanding of the pathophysiological mechanisms. Future investigations considering genetic variability and predisposing risk factors may strengthen the accuracy of predictive assessments and optimize intervention strategies for pregnancy toxemia in goats.

## Data Availability

All data are available within the manuscript.
